# Modern Management of Complex Regional Pain Syndrome: A Practical Evidence-Based Review for Hand and Upper Extremity Surgeons

**DOI:** 10.7759/cureus.113271

**Published:** 2026-07-24

**Authors:** Michael J Franco

**Affiliations:** 1 Plastic and Reconstructive Surgery, Cooper University Hospital, Camden, USA

**Keywords:** bisphosphonates, complex regional pain syndrome, hand surgery, mirror therapy, rehabilitation, stellate ganglion block

## Abstract

Complex regional pain syndrome (CRPS) is a debilitating pain disorder that most commonly develops following fractures, surgery, peripheral nerve injury, or other trauma to an extremity. Although substantial progress has been made in understanding its underlying neurobiology, CRPS remains difficult to diagnose and treat because of its heterogeneous clinical presentation and the absence of a single universally effective therapy. Patients often experience pain disproportionate to the inciting event, accompanied by sensory disturbances, autonomic dysfunction, edema, motor impairment, and trophic changes that collectively impair limb function and quality of life. For hand and upper-extremity surgeons, delayed recognition may result in prolonged disability, while premature diagnosis may obscure alternative conditions requiring surgical intervention.

This review synthesizes 22 full-text publications identified through a staged, Preferred Reporting Items for Systematic Reviews and Meta-Analyses (PRISMA)-style screening pathway of 1,471 PubMed records. Current literature consistently supports a multidisciplinary, function-centered treatment strategy. Clinical diagnosis using the Budapest Criteria remains the foundation of evaluation, while imaging and ancillary investigations are primarily used to exclude competing diagnoses. Early patient education, desensitization, edema management, and supervised rehabilitation represent the cornerstone of treatment. Pharmacologic therapies, including corticosteroids, bisphosphonates, neuropathic pain medications, and selected intravenous therapies, may facilitate functional recovery in carefully selected patients, although the certainty of evidence varies considerably. Sympathetic blockade, ketamine infusion, spinal cord stimulation, and dorsal root ganglion stimulation are best considered adjunctive therapies for patients with persistent symptoms despite comprehensive conservative management.

Current evidence favors a staged, multidisciplinary approach in which restoration of limb function serves as the primary therapeutic objective. Rehabilitation should begin as early as possible, while medications and interventional procedures should be used to facilitate participation in therapy rather than replace it. For hand and upper-extremity surgeons, prompt recognition, exclusion of alternative diagnoses, avoidance of unnecessarily prolonged immobilization, and coordinated multidisciplinary care remain the most important modifiable factors influencing patient outcomes.

## Introduction and background

Complex regional pain syndrome (CRPS) is a chronic pain disorder characterized by pain disproportionate to the inciting event and accompanied by variable combinations of sensory, vasomotor, sudomotor, motor, and trophic abnormalities. The syndrome most commonly develops following fractures, soft-tissue trauma, surgery, or peripheral nerve injury and frequently affects the distal upper extremity. Despite decades of investigation, CRPS remains one of the most challenging postoperative and post-traumatic complications encountered by hand surgeons because no individual clinical feature, imaging study, or laboratory investigation is sufficiently sensitive or specific to establish the diagnosis independently. Instead, diagnosis relies on careful clinical evaluation while systematically excluding competing explanations for the patient's symptoms [1-3].

Historically referred to as reflex sympathetic dystrophy and causalgia, CRPS is now recognized as a complex interaction among peripheral inflammation, autonomic dysfunction, central sensitization, immune activation, and maladaptive cortical plasticity. These overlapping mechanisms help explain the remarkable heterogeneity observed among patients and likely account for the inconsistent response to individual therapies reported throughout the literature. Rather than representing a single disease process, CRPS should be viewed as a syndrome involving multiple biological pathways that evolve over time and differ among patients [1-4].

Incidence figures should be interpreted with caution. Reported rates vary widely across studies because they depend heavily on which diagnostic criteria were applied, how intensively patients were surveilled, and how the study was designed. Distal radius fracture is the most frequently reported antecedent injury in the upper extremity [1,3]. CRPS following carpal tunnel release is uncommon but clinically meaningful, and most affected patients improve within one year. What matters clinically is less the population incidence than the recognition that any hand surgeon with a reasonable trauma and elective volume will encounter this condition.

For the practicing hand surgeon, CRPS presents unique diagnostic and therapeutic challenges. Patients recovering from distal radius fractures, tendon repairs, nerve reconstruction, crush injuries, or elective procedures such as carpal tunnel release frequently experience pain, edema, and stiffness during normal healing. Distinguishing expected postoperative recovery from evolving CRPS requires thoughtful clinical judgment and serial examination. Conversely, assigning a diagnosis of CRPS without excluding infection, persistent nerve compression, tendon pathology, hardware failure, vascular compromise, or fracture-related complications risks delaying appropriate surgical management. Maintaining a broad differential diagnosis is therefore essential throughout evaluation [1,3].

Although numerous pharmacologic, rehabilitative, and interventional therapies have been investigated, contemporary evidence demonstrates that few individual treatments consistently produce large or durable improvements when used in isolation. International guidelines, Cochrane reviews, systematic reviews, and randomized trials increasingly converge on a common principle: successful management depends upon coordinated multidisciplinary care centered on restoration of function rather than elimination of pain alone. Occupational and physical therapy, patient education, desensitization, and graded return to limb use remain the foundation of treatment, while medications, sympathetic blockade, infusion therapies, and neuromodulation should be considered adjunctive interventions designed to facilitate rehabilitation when pain prevents meaningful functional progress [1-5].

Unlike previous reviews that primarily catalog available interventions, the objective of this article is to provide a practical, evidence-based framework for the diagnosis and management of CRPS relevant to hand surgeons and upper-extremity specialists. Drawing upon contemporary international guidelines, systematic reviews, randomized controlled trials, and hand surgery-specific literature, we summarize current evidence while emphasizing clinical decision-making, multidisciplinary care, and realistic interpretation of therapeutic efficacy. Rather than identifying a single best treatment, this review advocates a staged approach in which treatment intensity is individualized according to disease severity, chronicity, functional impairment, and patient response.

## Review

This manuscript was designed as a Preferred Reporting Items for Systematic Reviews and Meta-Analyses (PRISMA)-style pathway review rather than a registered systematic review or de novo quantitative meta-analysis. The intent was to identify clinically useful articles capable of supporting a practical treatment pathway for adult CRPS, spanning diagnosis, early rehabilitation, pharmacologic therapy, intravenous therapies, sympathetic blockade, neuromodulation, prevention, and hand- and orthopaedic-specific considerations. The screening process is reported transparently and displayed in a PRISMA-style flow diagram so that readers can see exactly how the final evidence set was assembled, but no claim is made that this constitutes a systematic review with independent duplicate screening, formal risk-of-bias assessment, or quantitative synthesis.

Search strategy and screening pathway

A PubMed/MEDLINE search was performed using terms related to complex regional pain syndrome, CRPS, reflex sympathetic dystrophy, causalgia, treatment, rehabilitation, pharmacologic therapy, sympathetic blockade, neuromodulation, and prevention. The search identified 1,471 records. Because a single database was searched, no duplicate-removal step was required at the identification stage.

All 1,471 records underwent staged title and citation screening. During first-pass screening, 283 records were excluded as clearly irrelevant, most commonly because they were not CRPS-specific, did not address treatment or management, focused on unrelated chronic pain conditions, or represented low-yield mechanistic material without direct pathway relevance. The remaining 1,188 records were retained for staged triage, of which 170 were identified as likely relevant to CRPS treatment and 1,018 were deferred as lower-priority background material.

The 170 likely relevant records then underwent focused citation-level refinement. Seventy-five were excluded because they lacked direct CRPS-specific treatment data, consisted primarily of protocol, commentary, or letter material, addressed non-CRPS neuromodulation or general chronic pain populations, or were peripheral to the adult CRPS treatment pathway. Eighty-five core records advanced to abstract review, with 10 additional records retained as optional secondary background references.

Abstract-level screening of the 85 core records retained 67 as core included articles and 14 as optional background articles, and excluded 4. A full-text priority pool of 56 articles was defined, with a top-priority group of 50 and an essential first-download subset of 3

Literature review

The 22 unique full-text articles represent the subset of the screened literature judged most useful for constructing a practical treatment pathway. The set spans six clinical domains: diagnosis and care structure; functional restoration and rehabilitation; pharmacologic therapy; intravenous and infusion-based therapy; sympathetic blockade; and neuromodulation. It includes international guidelines and standards, Cochrane reviews and overviews of reviews, modality-specific systematic reviews and meta-analyses, randomized controlled trials, and hand- and orthopaedic-focused reviews. This constitutes a clinically focused synthesis rather than a claim that all relevant CRPS treatment literature was reviewed at full-text level.

Across all levels of evidence, a consistent theme emerges. Although numerous therapeutic interventions have demonstrated benefit in selected patient populations, no individual treatment has consistently demonstrated high-certainty disease-modifying effects across heterogeneous CRPS populations. Instead, the strongest agreement among contemporary guidelines is that treatment should be multidisciplinary, individualized, and centered upon restoration of function. Early rehabilitation, patient education, and avoidance of unnecessarily prolonged immobilization are repeatedly identified as fundamental principles regardless of disease stage, while pharmacologic and interventional therapies are most appropriately viewed as adjuncts that facilitate participation in rehabilitation rather than replacements for it [1-5].

This organizing principle serves as the framework for the remainder of this review. Each subsequent section examines a major treatment domain, summarizes the available evidence, discusses its practical application for hand surgeons and upper-extremity clinicians, and places individual therapies within a staged treatment strategy designed to maximize functional recovery while avoiding unnecessary interventions.

Review

Recognition and Diagnosis

Early recognition of CRPS remains one of the most important opportunities to improve patient outcomes. Although many patients initially present with pain, edema, stiffness, or altered sensation after trauma or surgery, the diagnosis should not be made on the basis of pain alone. Rather, CRPS is a clinical syndrome characterized by pain disproportionate to the inciting event, accompanied by variable combinations of sensory, vasomotor, sudomotor, motor, and trophic abnormalities. Because no laboratory test or imaging modality independently confirms the diagnosis, careful history-taking, serial physical examination, and exclusion of competing pathology remain essential components of evaluation [1-3].

The Budapest Criteria, developed by Harden and colleagues and now embedded within international practice guidelines, remain the accepted diagnostic standard and provide a reproducible clinical framework [1]. These criteria require continuing pain disproportionate to the inciting event, together with symptoms involving at least three of four clinical domains (sensory, vasomotor, sudomotor/edema, and motor/trophic) and objective findings in at least two domains during examination. Importantly, the diagnosis should only be established when no alternative condition better explains the patient's presentation [1,3]. The European Pain Federation standards articulated by Goebel and colleagues reinforce this point directly, emphasizing that diagnostic tests are not required to diagnose CRPS except when needed to exclude other conditions [3].

For hand surgeons, this final requirement is particularly important. Patients recovering from distal radius fractures, tendon repairs, peripheral nerve reconstruction, crush injuries, or elective hand procedures frequently develop pain, edema, stiffness, and weakness during normal healing. Likewise, postoperative infection, occult fracture displacement, tendon rupture, hardware complications, vascular insufficiency, persistent nerve compression, and adhesive scar formation may all mimic aspects of CRPS. Failure to recognize these competing diagnoses may delay definitive treatment, while delayed recognition of true CRPS may permit progressive pain, immobility, and disability. Consequently, diagnosis should remain dynamic throughout follow-up rather than being assigned during a single clinical encounter.

In practice, CRPS is frequently diagnosed late, often only after every competing diagnosis has been excluded across multiple visits. That caution is appropriate. But it carries a real cost, because rehabilitation gets deferred during precisely the window in which it is most likely to work. The diagnosis functions less like a switch that is flipped at a single visit and more like a photograph developing in a tray: the picture becomes recognizable gradually, and waiting for it to be fully sharp before acting wastes the time in which action matters most. Functional treatment should therefore begin during the diagnostic process rather than after it concludes.

Ancillary investigations should be viewed primarily as tools for excluding competing diagnoses rather than confirming CRPS. Plain radiographs may demonstrate regional osteopenia during later stages, characteristically appearing as patchy, mosaic-like demineralization in which small osteopenic areas sit juxtaposed against small areas of relatively preserved bone density. Radiographs are frequently unremarkable early in the disease course. Magnetic resonance imaging may identify bone marrow edema or soft tissue swelling, although its principal role is to exclude alternative pathology, including occult fracture, osteomyelitis, tendon injury, or avascular necrosis. Triple-phase bone scintigraphy has historically been advocated as a diagnostic adjunct; however, current international standards no longer recommend routine bone scintigraphy because of inconsistent diagnostic performance. Electrodiagnostic studies remain appropriate when traumatic peripheral nerve injury or persistent compressive neuropathy remains within the differential diagnosis [1,3].

From a practical perspective, clinicians should maintain a high index of suspicion when recovery deviates substantially from the expected postoperative course. Progressive allodynia, hyperalgesia, persistent edema, temperature asymmetry, color changes, sweating abnormalities, disproportionate stiffness, avoidance of limb use, and worsening rather than gradual improvement should all prompt careful reassessment.

Early intervention need not await absolute diagnostic certainty. This is the single most actionable point in the diagnostic section. Patient education, edema management, desensitization, and supervised rehabilitation carry minimal risk and should be initiated while continued evaluation excludes competing diagnoses. Nothing about starting therapy commits the surgeon to a diagnosis, and nothing about withholding it protects the patient.

Pathophysiology

Earlier concepts emphasized abnormal sympathetic nervous system activity as the principal mechanism responsible for persistent pain, which is how the term reflex sympathetic dystrophy came into wide use. Contemporary evidence instead supports a multifactorial model involving interactions among peripheral inflammation, autonomic dysregulation, immune activation, central sensitization, and maladaptive cortical reorganization. These overlapping mechanisms help explain both the heterogeneous clinical presentation of CRPS and the variable response to individual therapeutic interventions [1,4].

Following tissue injury, inflammatory mediators, including cytokines, neuropeptides, and growth factors, contribute to peripheral sensitization of nociceptive pathways. Neurogenic inflammation may produce warmth, erythema, edema, and hyperalgesia during early disease. Simultaneously, abnormal sympathetic activity contributes to disturbances in vasomotor regulation and sweating, resulting in the characteristic temperature and color asymmetry observed in many patients. As the disorder progresses, persistent nociceptive input may promote central sensitization, reducing pain thresholds and amplifying responses to otherwise innocuous stimuli such as light touch or joint movement. The nervous system behaves less like a wire faithfully carrying a signal and more like an amplifier whose gain has been turned up and left there.

Functional neuroimaging and neurophysiologic studies have also demonstrated cortical reorganization involving somatosensory and motor regions of the brain. These findings provide a biologic rationale for rehabilitation strategies such as graded motor imagery, laterality recognition, and mirror therapy, which aim to normalize cortical processing while gradually restoring movement. Although the precise mechanisms remain incompletely understood, these observations reinforce the concept that CRPS cannot be adequately explained by peripheral pathology alone and likely represents a dynamic interaction between peripheral injury and central nervous system adaptation [6-10].

Current pathophysiologic understanding also explains why no single therapeutic intervention consistently produces reliable disease resolution. Anti-inflammatory medications primarily target peripheral inflammatory mechanisms, sympathetic blockade influences autonomic dysfunction, neuromodulation alters central pain processing, and rehabilitation addresses motor dysfunction, cortical reorganization, and functional restoration. Each therapy is aimed at a different point along the pathway. Because individual patients likely exhibit different contributions from each mechanism, a treatment that works well in one patient may be aimed at a mechanism that is not driving disease in the next. This is the biological argument for multidisciplinary treatment strategies that combine rehabilitation with carefully selected pharmacologic and interventional therapies.

Clinical pearl. Modern CRPS should be viewed as a multisystem pain syndrome rather than a sympathetically mediated disorder alone. Understanding its multifactorial pathophysiology helps explain why multidisciplinary treatment consistently outperforms reliance on any single medication or procedure and reinforces the importance of individualized, function-centered care.

Functional Restoration: The Cornerstone of Modern CRPS Management

Despite significant advances in pharmacologic therapy, neuromodulation, and interventional pain management, the most consistent message emerging from contemporary CRPS literature is that restoration of function remains the primary therapeutic objective. International guidelines, systematic reviews, and randomized trials consistently emphasize that treatment should focus on improving limb use, reducing disability, and preventing long-term functional decline rather than simply reducing pain intensity. Although pain relief is an important component of management, pain reduction alone rarely translates into meaningful recovery unless accompanied by progressive restoration of motion, strength, dexterity, and confidence in using the affected extremity [1-5].

This philosophy represents a fundamental shift from earlier treatment paradigms. Historically, management often focused on interrupting sympathetic activity or pursuing increasingly invasive interventions in an effort to eliminate pain. Stanton-Hicks and colleagues reframed the problem in 1998 around motivation, mobilization, desensitization, and self-management, with medications, blocks, and neuromodulation used to facilitate progress rather than to replace rehabilitation [2]. The fifth edition of the practical guidelines maintains the same clinical logic: treatment must be individualized and interdisciplinary, with escalating support when pain, disability, distress, or complications prevent progress [1]. Medications, sympathetic blockade, infusion therapies, and neuromodulation are therefore best understood as tools that buy access to rehabilitation, not as definitive treatments in isolation [1,2].

An honest reading of the Cochrane evidence complicates this picture in a way worth stating plainly. Smart and colleagues found that the evidence base for physiotherapy interventions in CRPS remains limited and uncertain [5]. This does not mean therapy is unimportant; it means the literature does not support confident claims that any one protocol is uniformly effective. Rehabilitation is the backbone of care, but the specific protocol should be individualized rather than prescribed from a manual.

Patient Education and Expectation Setting

The initial patient encounter provides an important opportunity to establish realistic expectations and reduce fear associated with persistent pain. Many patients believe that movement will worsen tissue injury, particularly following fractures or surgery. This fear frequently results in protective guarding, avoidance of limb use, progressive stiffness, and further functional decline. Early education should therefore emphasize that CRPS is a genuine pain disorder involving abnormal nervous system processing rather than ongoing structural damage. Patients should understand that gradual movement, guided rehabilitation, and progressive functional use are essential components of recovery [1-3].

Equally important is reassuring patients that improvement often occurs gradually over weeks or months rather than days. Establishing functional goals, such as improving finger motion, restoring grip strength, resuming self-care activities, or returning to work, helps shift attention from pain scores toward meaningful recovery. This patient-centered approach is repeatedly emphasized within contemporary practice guidelines and forms the foundation of successful multidisciplinary management [1-3].

Occupational and Physical Therapy

Occupational and physical therapy remain the cornerstone of CRPS treatment regardless of disease severity [2,5]. Therapy should begin as soon as medically appropriate and should be individualized according to the patient's symptoms, stage of recovery, and underlying pathology. Early interventions typically emphasize edema reduction, active range-of-motion exercises, tendon gliding, joint mobilization within patient tolerance, and progressive functional use of the extremity.

One of the most important principles is the avoidance of unnecessarily painful rehabilitation. Aggressive passive stretching may reinforce pain behaviors and reduce patient participation. Instead, therapy should progress gradually using active movement, graded exposure, and task-oriented exercises that encourage normal limb use while minimizing fear-avoidance. Frequent reassessment allows therapists to modify treatment intensity according to patient response while maintaining steady progress toward functional goals.

For patients recovering from fractures or surgery, rehabilitation should also account for procedure-specific precautions. Tendon repairs, fracture fixation, peripheral nerve reconstruction, and ligament reconstruction each require individualized protocols that balance tissue protection with avoidance of unnecessarily prolonged immobilization. Close communication between surgeon and therapist is essential throughout treatment.

Desensitization and Edema Management

Persistent hypersensitivity frequently limits participation in rehabilitation and contributes substantially to disability. Desensitization techniques are therefore introduced early in treatment and generally progress from gentle tactile stimulation toward increasingly complex sensory input. Common strategies include exposure to fabrics of varying texture, vibration, massage, immersion techniques, and graded sensory discrimination exercises.

Edema management is equally important because persistent swelling contributes to stiffness, pain, reduced tendon excursion, and diminished hand function. Elevation, compression garments, manual edema mobilization, active muscle pumping exercises, and appropriate splinting may all be incorporated according to individual patient needs. Although high-quality comparative evidence remains limited, these interventions are widely accepted components of multidisciplinary rehabilitation and are consistently incorporated into contemporary treatment guidelines [1-5].

Graded Motor Imagery and Mirror Therapy

Among rehabilitation interventions, graded motor imagery (GMI) has received considerable attention because it directly addresses maladaptive cortical reorganization believed to contribute to persistent pain and impaired motor control. The sequential GMI program described by Moseley begins with left-right limb laterality recognition, progresses to imagined movement, and culminates in mirror therapy [6]. This structured progression is designed to activate cortical motor networks while minimizing pain associated with direct movement. Moseley's 2005 trial suggested that the order of these steps matters, supporting sequential activation of cortical motor networks rather than mere sustained attention to the affected limb [7]. The program works something like tuning an instrument before playing it rather than forcing music out of strings that are badly out of tune.

Randomized trials have demonstrated improvements in pain, swelling, and disability among selected patients with chronic upper-extremity CRPS, although study populations remain small and methodological heterogeneity limits definitive conclusions [6,8]. Subsequent systematic reviews acknowledge these encouraging findings while emphasizing the need for larger, higher-quality trials [5]. Graded motor imagery should therefore be viewed as a valuable component of rehabilitation, particularly when direct movement remains poorly tolerated, rather than a universally effective treatment applicable to every patient.

Mirror therapy provides an accessible extension of graded motor imagery by using visual feedback from the contralateral extremity to modify cortical processing. Many therapists incorporate mirror therapy into home exercise programs because it is inexpensive, safe, and easily reproduced outside the clinical setting. Although the overall certainty of evidence remains moderate to low, the minimal associated risk and potential functional benefit support its inclusion within a comprehensive rehabilitation program.

Restoring Meaningful Function

Perhaps the most important objective of rehabilitation is restoration of meaningful participation in daily life. Improvements in pain scores alone do not necessarily translate into successful recovery if patients remain unable to perform activities of daily living, return to employment, participate in family responsibilities, or resume recreational pursuits. Consequently, therapy should increasingly emphasize functional tasks rather than isolated joint motion as recovery progresses.

Return-to-work planning should begin early whenever possible. For many patients, graduated work restrictions, adaptive equipment, ergonomic modifications, and close communication with employers facilitate successful reintegration while avoiding prolonged disability. Hand therapists frequently play an essential role in identifying workplace barriers and developing individualized strategies that allow progressive return to meaningful activity.

The Role of the Hand Surgeon

For surgeons managing patients with CRPS, rehabilitation should not be viewed simply as a referral but rather as the central component of treatment. Surgical follow-up visits provide repeated opportunities to reinforce therapy participation, encourage progressive limb use, monitor recovery, and identify barriers limiting functional improvement. Pharmacologic therapy, sympathetic blockade, or referral to pain specialists should be considered when pain prevents meaningful engagement in rehabilitation rather than as substitutes for therapy itself.

This rehabilitation-centered philosophy is perhaps the most important practical message emerging from contemporary literature. Across international guidelines, systematic reviews, Cochrane analyses, and randomized trials, individual therapies demonstrate variable efficacy. In contrast, restoration of function remains the consistent therapeutic objective linking every stage of modern CRPS management.

Pharmacologic Management

Pharmacologic therapy remains an important adjunct in the multidisciplinary management of CRPS, yet no medication has consistently demonstrated high-certainty disease-modifying benefit across heterogeneous patient populations. Contemporary systematic reviews and international guidelines increasingly emphasize that medications should be individualized according to disease stage, symptom severity, and functional impairment, with the primary objective of enabling participation in rehabilitation rather than achieving complete pain resolution [1-5]. The principal medication categories, their evidence base, and their practical role are summarized in Table 1. Each subsection below separates what the evidence shows from what we recommend doing with it.

Bisphosphonates

Evidence. Among currently available pharmacologic therapies, bisphosphonates possess the strongest evidence supporting clinical benefit in selected patients. Their proposed mechanism extends beyond inhibition of osteoclastic activity and may involve modulation of inflammatory pathways and reduction of abnormal bone turnover observed in early CRPS. Fassio and colleagues found the most consistent pharmacologic signal for bisphosphonates in adult CRPS-I, with a smaller signal for ketamine and less clear benefit for other medication classes [8]. The neridronate trial reported by Varenna and colleagues was strongly positive, and notably enrolled patients with acute CRPS-I, positive bone scintigraphy, and short symptom duration [10]. That entry profile remains the most clearly defined responder phenotype anywhere in the CRPS pharmacologic literature.

More recent evidence has introduced greater caution. The updated meta-analysis by Ferraro and colleagues continues to demonstrate potential short-term pain improvement but emphasizes low certainty of evidence, substantial clinical heterogeneity, and a probable increase in adverse events, with uncertain medium- and long-term effects [9]. Evidence supporting durable long-term benefit remains limited [4,9].

Practical recommendation. Bisphosphonates should neither be considered universal first-line therapy nor dismissed entirely. They appear most appropriate for carefully selected patients with relatively early CRPS-I, particularly when inflammatory features or regional bone metabolic changes predominate,and should be initiated only after individualized consideration of contraindications, renal function, dental status, adverse effects, and disease stage.

Neuropathic Pain Medications

Evidence. Gabapentinoids, tricyclic antidepressants, serotonin-norepinephrine reuptake inhibitors, and other neuropathic pain medications are commonly prescribed for CRPS despite relatively limited disease-specific evidence. Their use is largely extrapolated from the broader neuropathic pain literature rather than supported by large randomized CRPS trials. Available systematic reviews have not demonstrated consistent superiority of any single medication class, and treatment response varies considerably among individuals [8].

Practical recommendation. Given their relatively favorable safety profile and widespread familiarity, neuropathic pain medications remain reasonable adjuncts for patients with prominent burning pain, dysesthesia, allodynia, or sleep disturbance. Selection should be individualized according to comorbidities, adverse-effect profiles, and previous treatment response. They are best regarded as adjunctive therapy supporting rehabilitation rather than disease-modifying treatment.

Ketamine

Evidence. Ketamine has attracted interest because of its antagonism of N-methyl-D-aspartate (NMDA) receptors and its potential to reduce central sensitization. Connolly and colleagues concluded that high-quality evidence was lacking and that overall support for ketamine was weak [11]. Small clinical studies and systematic reviews suggest that intravenous infusion may reduce pain in carefully selected patients with refractory disease, but improvements in functional outcomes have been less consistent [11,12]. The literature is limited by small sample sizes, heterogeneous infusion protocols, variable duration of benefit, and inconsistent reporting of adverse events.

Practical recommendation. Ketamine is a pain-specialist intervention. Reserve it for carefully selected patients whose persistent pain prevents meaningful participation in rehabilitation despite optimized conservative management, and manage it within experienced multidisciplinary pain programs. Administration requires specialized monitoring given psychotropic side effects and cardiovascular considerations.

Intravenous Therapies

Evidence. Several additional intravenous therapies, including lidocaine, magnesium, intravenous immunoglobulin, and intravenous bisphosphonates, have been investigated. Xu and colleagues found evidence supporting selected intravenous approaches while calling for higher-quality efficacy, safety, and cost-effectiveness data [12]. Systematic reviews consistently conclude that overall evidence quality remains low because of limited patient numbers, methodological heterogeneity, and inconsistent outcome reporting [11-14].

Practical recommendation. No infusion therapy can be recommended for routine use. Consider these interventions on an individual basis after failure of standard multidisciplinary management, preferably within specialized pain centers familiar with their administration.

Corticosteroids

Evidence. Inflammation appears to play an important role during the early stages of CRPS, providing a biologic rationale for corticosteroid therapy in carefully selected patients. Broad reviews of randomized trials support short courses of oral steroids in selected contexts while emphasizing the variable quality and heterogeneity of the underlying trials [15-17]. Clinical experience and several randomized studies suggest that short courses may reduce pain, edema, and stiffness during the acute inflammatory phase, particularly when treatment is initiated soon after symptom onset. The available literature remains heterogeneous with respect to patient selection, dosing strategies, treatment duration, and outcome measures, which limits definitive conclusions regarding optimal protocols.

Practical recommendation. Consider a short corticosteroid course during the early inflammatory phase in patients with warmth, edema, inflammatory skin changes, and rapidly progressive stiffness. Prolonged corticosteroid therapy has not demonstrated consistent benefit and should generally be avoided because of well-recognized systemic adverse effects. Steroid treatment should complement rather than delay aggressive rehabilitation.

Emerging and Other Pharmacologic Therapies

Numerous additional medications, including calcitonin, topical dimethyl sulfoxide, nonsteroidal anti-inflammatory drugs, antioxidants, and other anti-inflammatory agents, have been studied with variable results. While some early investigations suggested potential benefit, subsequent systematic reviews have not demonstrated sufficiently consistent evidence to recommend routine use across the broader CRPS population [15-19].

Opioid therapy should not be considered a disease-modifying treatment. Although short-term use may occasionally be necessary during severe exacerbations, prolonged opioid therapy has not demonstrated consistent functional benefit and should be approached cautiously because of well-recognized risks of tolerance, dependence, and opioid-induced hyperalgesia.

Practical Pharmacologic Approach

Pharmacologic therapy should be individualized according to disease stage, symptom profile, comorbidities, and response to rehabilitation. Current evidence supports a pragmatic sequence: a short course of corticosteroids in carefully selected patients with early inflammatory CRPS; bisphosphonate therapy in selected patients with early CRPS-I after individualized risk assessment; neuropathic pain medications to improve sleep, neuropathic symptoms, and tolerance of rehabilitation; and ketamine infusion or other intravenous therapies reserved for refractory patients within multidisciplinary pain programs.

Throughout, prolonged escalation of pharmacologic therapy in the absence of measurable functional improvement should be avoided. The question at every visit is not whether the pain score moved but whether the patient is doing more with the hand than they were a month ago. If pharmacologic therapy is not facilitating rehabilitation or improving participation in activities of daily living, the treatment strategy needs reconsideration rather than another agent added to it.

**Figure 1:**
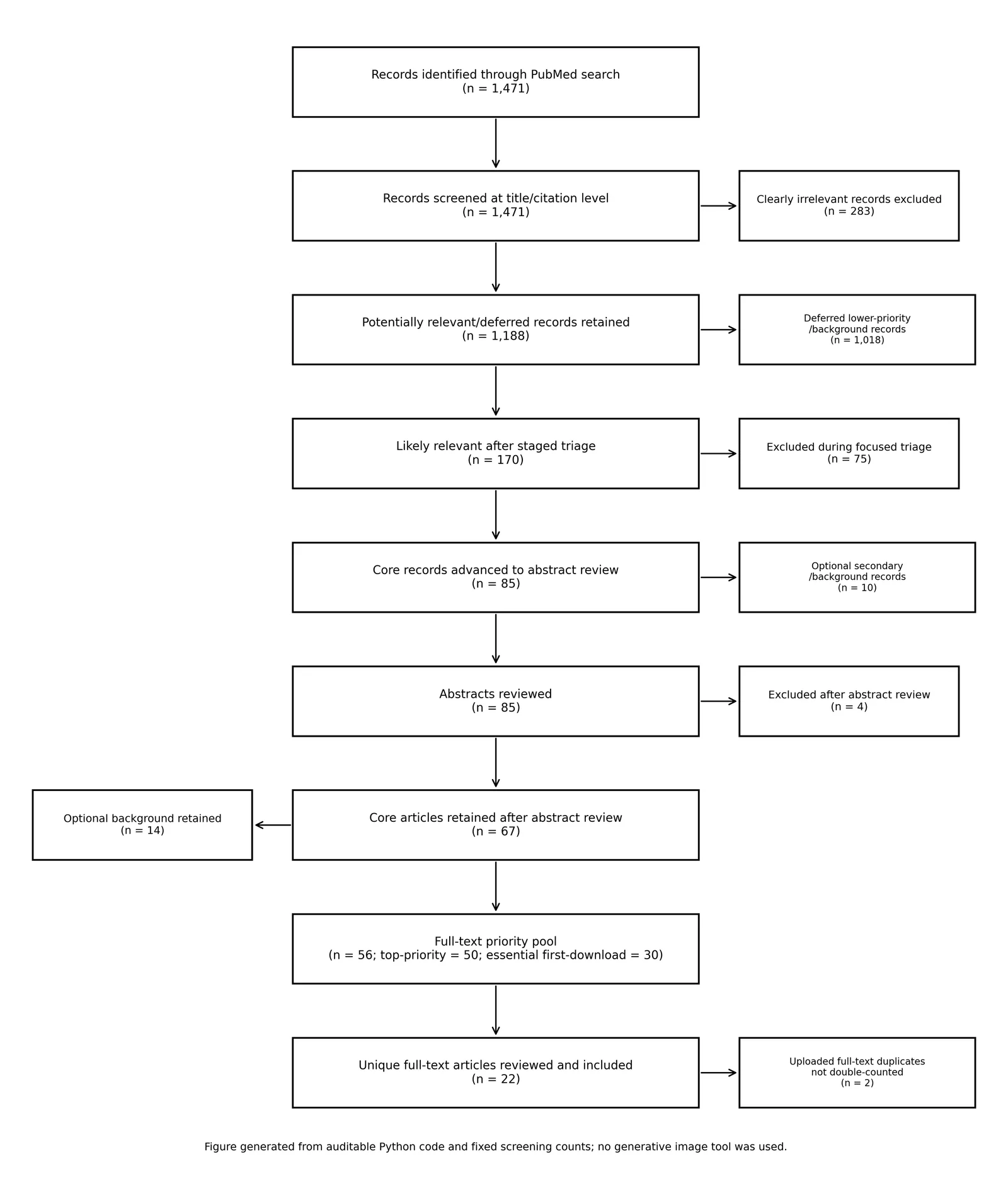
PRISMA-style study selection pathway Records identified through PubMed (n = 1,471); first-pass exclusions (n = 283); staged triage identifying likely relevant records (n = 170) with lower-priority material deferred (n = 1,018); focused triage exclusions (n = 75); abstracts reviewed (n = 85) with exclusions (n = 4); full-text priority pool (n = 56); PDFs retrieved (n = 24) with duplicates logged (n = 2); unique full-text articles included (n = 22). The figure was generated from an auditable Python script using fixed screening counts from the project log. No generative image tool was used. PRISMA: Preferred Reporting Items for Systematic Reviews and Meta-Analyses.

Twenty-four full-text PDFs were retrieved and reviewed; two were duplicates and were logged without double-counting, leaving 22 unique full-text articles. No unique full-text article was excluded after full-text review. These 22 articles constitute the reference set for this manuscript. The complete pathway is displayed in Figure 1.

Inclusion Criteria and Rationale for Database Selection

Inclusion was limited to English-language, full-text publications addressing adult populations, with preference given to international practice guidelines, Cochrane reviews, systematic reviews with or without meta-analysis, and randomized controlled trials. Reference lists of included articles were hand-searched for additional relevant citations. Priority was given to publications from 2013 onward, with older references retained when they represented foundational or still-authoritative work.

PubMed was used as the sole search database by design rather than by omission. Cochrane Library records identified during preliminary searching were, with rare exception, already indexed in PubMed and added little unique yield. Embase was intentionally excluded because access requires an institutional subscription and, in most settings, librarian support. A search strategy that any clinician can reproduce from a laptop using a freely available database was judged more valuable for a practical, pathway-oriented review than a marginally more sensitive strategy that most readers could not replicate. This is a genuine trade-off, not a costless one, and it is stated again in the Limitations section.

Data Extraction

For each included full text, extracted fields included citation information, study type, population, CRPS subtype when reported, intervention or treatment category, comparator, principal outcomes, key findings, limitations, and pathway relevance. The master extraction sheet was maintained as an audit file so that each manuscript claim can be traced to a specific source. It is available to editors and readers on request.

Assessment of Evidence Quality

Evidence quality designations in Tables 1, 2 are not the product of an independent GRADE assessment performed by the author. They reproduce the certainty ratings reported in the source Cochrane reviews, systematic reviews, and international guidelines cited in each row. Where a source did not report a formal certainty rating, the designation reflects the author's interpretation of study design, sample size, and consistency of findings, and should be read as such. No new risk-of-bias scoring was performed for individual trials beyond extracting the risk-of-bias and certainty judgments already reported within the included reviews.

**Table 1:** Summary of pharmacologic therapies for complex regional pain syndrome Quality-of-evidence designations reproduce certainty ratings reported in the cited source reviews and guidelines. Where a source did not report a formal certainty rating, the designation reflects the author's interpretation of study design, sample size, and consistency of findings. No independent GRADE assessment was performed. CRPS: complex regional pain syndrome, GRADE: Grading of Recommendations Assessment, Development, and Evaluation.

Therapy	Primary role	Quality of evidence	Practical recommendation
Corticosteroids	Early inflammatory CRPS	Moderate	Consider a short course in carefully selected patients with early inflammatory disease.
Bisphosphonates	Early CRPS-I	Moderate (strongest medication evidence); low certainty in updated meta-analysis	Consider in selected patients after individualized risk-benefit assessment.
Neuropathic pain medications	Symptom control	Low-Moderate	Adjunctive therapy to improve tolerance of rehabilitation and symptom control.
Ketamine infusion	Refractory CRPS	Low	Reserve for multidisciplinary pain programs after failure of conservative management.
Intravenous therapies	Selected refractory patients	Low	Not recommended as routine first-line treatment.
Opioids	Acute rescue only	Limited	Avoid prolonged therapy whenever possible because functional benefit is limited.

**Table 2:** Summary of interventional therapies for complex regional pain syndrome Quality-of-evidence designations reproduce certainty ratings reported in the cited source reviews and guidelines. Where a source did not report a formal certainty rating, the designation reflects the author's interpretation. No independent GRADE assessment was performed. CRPS: complex regional pain syndrome, GRADE: Grading of Recommendations Assessment, Development, and Evaluation.

Intervention	Primary role	Quality of evidence	Practical recommendation
Stellate ganglion block	Upper-extremity CRPS	Low-Moderate	Consider when pain limits participation in rehabilitation.
Lumbar sympathetic block	Lower-extremity CRPS	Low	Individualized use within multidisciplinary care.
Continuous regional anesthesia	Severe pain preventing rehabilitation	Low	Selected patients undergoing intensive rehabilitation.
Ketamine infusion	Refractory CRPS	Low	Pain-specialist intervention after failure of conservative treatment.
Spinal cord stimulation	Chronic refractory CRPS	Moderate	Consider after comprehensive multidisciplinary treatment has failed; pain relief exceeds functional gain.
Dorsal root ganglion stimulation	Focal refractory CRPS	Moderate	Highly selected patients; strongest evidence currently exists for lower-extremity disease.

Figure Generation and Auditability

The PRISMA-style flow diagram was generated from a Python script that stores the screening counts in a fixed dictionary and draws the figure using matplotlib (Figure 1). This approach was chosen so that the figure can be regenerated and audited from code rather than accepted on trust. No generative image tool was used. The script is provided in the Appendices.

Interventional Management

Interventional pain procedures occupy an important but carefully defined role. While rehabilitation remains the cornerstone of treatment, selected interventions may provide meaningful pain reduction that allows patients to participate more effectively in therapy. Contemporary evidence does not support routine use of interventional procedures as definitive stand-alone therapy [1-5]. The major interventional options are summarized in Table 2.

Timing is as important as selection. Patients who continue to demonstrate progressive functional improvement through rehabilitation alone generally do not require invasive procedures. Conversely, individuals whose pain prevents meaningful participation in therapy despite optimized conservative management may benefit from referral to a multidisciplinary pain service.

Sympathetic Blockade

Evidence. Sympathetic blockade has historically represented one of the most frequently performed interventions for CRPS, particularly stellate ganglion block for upper-extremity disease and lumbar sympathetic block for lower-extremity involvement. The Cochrane review by O'Connell and colleagues found low- to very-low-certainty evidence and did not identify strong support for local anesthetic sympathetic blockade as an effective stand-alone intervention [13]. These findings reflect small study populations, heterogeneous patient selection, inconsistent procedural techniques, and variable outcome measures. A more recent stellate ganglion block meta-analysis by Tian and colleagues reported modest improvements in pain intensity among selected patients with upper-extremity CRPS, although substantial heterogeneity remains and larger randomized trials are needed [14].

Practical recommendation. The primary value of sympathetic blockade may be temporary pain reduction sufficient to permit more effective participation in rehabilitation. Think of it as prying open a window rather than fixing the house. Consider it for carefully selected patients whose pain substantially limits therapy participation, particularly when pain rather than stiffness or fear-avoidance is the dominant barrier, and integrate it into a multidisciplinary plan rather than performing it as isolated therapy.

Regional Anesthesia

Evidence. Continuous regional anesthesia and repeated peripheral nerve blocks have been described for selected patients with severe CRPS, particularly when pain prevents active movement or postoperative rehabilitation. By temporarily reducing nociceptive input, regional anesthesia may create an opportunity for intensive therapy. High-quality evidence supporting prolonged regional anesthetic techniques remains limited; most published literature consists of small series or narrative reviews rather than large randomized trials [12,15].

Practical recommendation. Consider in selected patients when severe pain prevents rehabilitation, provided treatment is coordinated with intensive occupational or physical therapy. Achieving temporary analgesia without immediately using that window for functional rehabilitation is unlikely to produce long-term benefit.

Ketamine Infusion

Evidence. Systematic reviews suggest ketamine infusion may provide short-term pain reduction in selected patients; improvements in function are less consistent, and a durable benefit remains uncertain [11,12]. Administration requires specialized monitoring because of psychotropic effects, cardiovascular considerations, and variability in dosing protocols.

Practical recommendation. An option for carefully selected patients managed within experienced multidisciplinary pain programs after failure of conventional rehabilitation and pharmacologic therapy.

Spinal Cord Stimulation

Evidence. Spinal cord stimulation remains the most extensively investigated neuromodulation technique for chronic refractory CRPS. The two-year follow-up of Kemler's randomized trial demonstrated improved pain and global perceived effect with stimulation plus physical therapy, but without clinically important functional improvement [20]. This gap between pain relief and functional gain recurs throughout the neuromodulation literature and deserves explicit mention during counseling. A later systematic review found low-frequency stimulation superior to conventional therapy or placebo stimulation, but evidence remains limited regarding whether newer parameters, including burst and high-frequency paradigms, outperform conventional techniques [21]. Device-related complications, revision procedures, and declining effectiveness over time should also be discussed [20,21].

Practical recommendation. Consider only after failure of comprehensive conservative treatment and multidisciplinary evaluation, with successful trial stimulation, appropriate patient selection, and realistic expectation setting.

Dorsal Root Ganglion Stimulation

Evidence. Dorsal root ganglion stimulation allows more focal stimulation of painful dermatomes while potentially reducing unwanted paresthesia. The ACCURATE randomized comparative trial reported by Deer and colleagues demonstrated higher treatment success rates with DRG stimulation than conventional spinal cord stimulation among patients with chronic lower-extremity CRPS or causalgia at both 3 and 12 months [22]. Most available evidence involves lower-extremity disease rather than upper-extremity CRPS, and long-term comparative outcomes remain limited.

Practical recommendation. Appropriate for highly selected patients evaluated within specialized neuromodulation programs. Extrapolation to hand and upper-extremity CRPS should be undertaken cautiously while upper-extremity-specific studies are awaited.

Multidisciplinary Escalation of Care

The most important lesson from the contemporary literature is that interventional procedures should support rehabilitation rather than replace it. Escalation should occur when pain, psychological distress, or autonomic dysfunction prevents meaningful participation in functional restoration despite appropriate conservative management. Referral to an interdisciplinary pain program should be considered when patients demonstrate persistent disability despite optimized rehabilitation, pharmacologic therapy, and surgeon-directed care.

Several practical principles follow. Interventional procedures should facilitate rehabilitation rather than replace it. Sympathetic blocks provide the greatest value when they create a temporary window for functional therapy. Pain reduction alone should not be considered a successful treatment unless accompanied by measurable improvement in function. And careful patient selection ultimately matters more than the choice of any individual procedure.

Prevention and Postoperative Considerations for Hand and Upper Extremity Surgeons

Although considerable attention has been directed toward treatment of established CRPS, prevention remains the most effective strategy for reducing long-term disability. Hand surgeons occupy a unique position because they frequently manage patients during the earliest stages of injury and postoperative recovery, when surgical technique, rehabilitation, patient education, and coordinated follow-up may reduce the risk of persistent pain and functional decline. Saed and colleagues emphasized early diagnosis, multidisciplinary care, and trauma-specific management in the orthopaedic setting [18]. While no intervention completely eliminates the development of CRPS, contemporary evidence supports several practical principles [1-5]. These recommendations are consolidated in Table 3.

**Table 3:** Practical clinical recommendations for hand surgeons CRPS: complex regional pain syndrome.

Clinical situation	Recommended approach
Suspected CRPS after surgery	Apply the Budapest Criteria while excluding infection, tendon rupture, hardware failure, vascular compromise, and persistent nerve compression.
Early inflammatory CRPS	Begin supervised hand therapy immediately; consider a short corticosteroid course in selected patients.
Persistent edema and hypersensitivity	Emphasize edema control, desensitization, and progressive functional use.
Pain limiting rehabilitation	Consider adjunctive medications and referral for sympathetic blockade or pain management.
Failure of conservative management	Obtain multidisciplinary evaluation and consider neuromodulation in carefully selected patients.
Primary treatment goal	Restore function, independence, return to work, and meaningful limb use rather than focusing solely on pain reduction.

Identifying Patients at Increased Risk

CRPS most commonly develops following fractures, crush injuries, tendon lacerations, peripheral nerve injury, or upper-extremity surgery. Distal radius fractures represent one of the most frequently reported antecedent injuries and deserve particular attention during postoperative follow-up [1,3]. Patients experiencing prolonged immobilization, severe postoperative pain disproportionate to expected recovery, persistent edema, or delayed restoration of motion should undergo careful serial evaluation for evolving CRPS while alternative surgical complications are simultaneously excluded.

Proposed risk factors include female sex, increasing age, osteoporosis, smoking, migraine, anxiety, depression, and prior CRPS. In the carpal tunnel literature specifically, Sousa and colleagues identified female sex, dominant-hand surgery, immobilization, and tourniquet time as associated factors, while emphasizing that the supporting evidence remains limited [19]. It is worth stating plainly what the literature does not provide: the sources synthesized here do not report pooled predictive values, sensitivities, specificities, or odds ratios for these variables, and no individual variable or combination has been validated as a clinically useful predictive instrument. Preoperative risk stratification should therefore not be attempted. Postoperative surveillance based on clinical trajectory remains the more reliable approach, and the trajectory is a far better signal than the demographic profile.

Surgical Technique and Soft-Tissue Management

Meticulous surgical technique remains fundamental to minimizing postoperative complications that may contribute to prolonged pain and delayed rehabilitation. Gentle soft-tissue handling, preservation of vascularity, stable fracture fixation when indicated, meticulous nerve protection, and careful hemostasis all support early postoperative mobilization. Although no surgical technique has been proven to prevent CRPS directly, minimizing unnecessary tissue trauma and avoiding secondary complications reduces barriers to rehabilitation.

Persistent postoperative pain should never be attributed to CRPS without careful evaluation for infection, tendon injury, hardware irritation, compartment syndrome, vascular compromise, recurrent compression neuropathy, or fracture-related complications. Continued reassessment remains particularly important during the early postoperative period when multiple conditions may produce overlapping findings.

Early Mobilization

Perhaps the most consistent recommendation emerging from contemporary guidelines is avoidance of unnecessarily prolonged immobilization. Temporary protection remains essential and appropriate following fractures, tendon repairs, ligament reconstruction, and peripheral nerve surgery; the concern is not immobilization itself but immobilization extended past the point of surgical necessity, which contributes to stiffness, edema, muscle atrophy, altered sensorimotor integration, and fear of movement. Whenever surgical stability permits, controlled early mobilization should begin under the guidance of an experienced hand therapist [1-5].

Early rehabilitation should emphasize edema reduction, active range of motion, tendon gliding, scar management, desensitization, and progressive functional use of the extremity. Rehabilitation protocols must always respect procedure-specific precautions; restoration of safe movement should remain an explicit objective from the beginning of treatment rather than a delayed consideration after pain improves.

Hand Therapy and Patient Education

Communication between surgeon, therapist, and patient is one of the most important determinants of successful postoperative recovery. Patients should understand that temporary discomfort during rehabilitation does not necessarily indicate structural injury and that progressive use of the extremity is essential for recovery. Fear-avoidance behaviors frequently develop following traumatic injuries and may contribute substantially to persistent disability if not recognized early.

Hand therapists play a central role by providing edema management, scar mobilization, sensory re-education, desensitization techniques, graded strengthening, and task-specific functional training. Frequent communication between therapist and surgeon allows early recognition of unexpected deterioration, plateauing recovery, or findings suggesting alternative diagnoses requiring further investigation.

Vitamin C

Vitamin C has received considerable attention as a potential prophylactic intervention following distal radius fracture. Earlier randomized trials suggested that supplementation might reduce CRPS incidence, leading to widespread adoption. More recent systematic reviews and updated evidence have produced less consistent conclusions, with several analyses questioning both the magnitude and the certainty of any protective effect [4,15].

Given its favorable safety profile and low cost, some surgeons continue to recommend supplementation after distal radius fracture despite this uncertainty. Patients should be counseled that any potential benefit appears modest and that vitamin C should not replace established preventive measures such as appropriate fracture management, edema control, and early rehabilitation.

Tourniquet Use

Tourniquet-related ischemia has occasionally been proposed as a contributor to postoperative pain syndromes, and tourniquet time appears among the associated factors reported in the carpal tunnel literature [19]. However, convincing evidence directly linking routine tourniquet use to CRPS development remains lacking, and current literature does not support modification of standard tourniquet practice solely to prevent CRPS. Surgeons should continue to employ accepted principles of tourniquet safety, including appropriate cuff selection, inflation pressure, and minimization of ischemia time whenever feasible.

Postoperative Surveillance

Recognition of CRPS often depends upon identifying deviations from the expected recovery trajectory rather than isolated examination findings. Routine postoperative visits should include assessment of pain progression, edema, active range of motion, temperature asymmetry, skin color changes, sweating abnormalities, hypersensitivity, and willingness to use the affected extremity. Progressive deterioration despite technically successful surgery should prompt careful reassessment rather than reassurance alone.

When CRPS is suspected, early multidisciplinary intervention should begin promptly while alternative diagnoses continue to be evaluated. Delaying rehabilitation until diagnostic certainty is achieved may unnecessarily prolong disability and contribute to progressive functional decline.

Practical Recommendations for Hand Surgeons

Several practical principles emerge consistently. Restore stable anatomy while minimizing unnecessary soft-tissue trauma. Begin supervised rehabilitation as early as procedure-specific healing permits, avoiding unnecessarily prolonged immobilization. Treat persistent postoperative edema and hypersensitivity aggressively. Maintain a broad differential diagnosis throughout recovery so that competing surgical complications are not overlooked. Refer early for multidisciplinary management when rehabilitation progress plateaus because of pain. And measure success by restoration of meaningful function rather than pain reduction alone.

Ultimately, prevention of CRPS is best viewed as an extension of thoughtful postoperative care rather than a separate therapeutic intervention. Careful surgery, structured rehabilitation, patient education, and vigilant postoperative surveillance remain the most effective strategies available.

Limitations of Current Evidence

Limitations of the literature. CRPS populations are markedly heterogeneous with respect to inciting injury, disease duration, diagnostic criteria applied, affected limb, subtype, and psychosocial context. Outcome measurement is inconsistent; pain intensity remains the most frequently reported endpoint despite broad agreement that function is the more meaningful objective, and validated upper-extremity functional instruments are applied inconsistently. Most randomized trials are small, single-center, and underpowered for functional endpoints or subgroup analysis. Heterogeneity in dosing, procedural technique, treatment duration, and follow-up interval precludes meaningful pooled estimates across many domains. Much of the neuromodulation evidence, including the ACCURATE trial, derives from lower-extremity disease, and extrapolation to hand and upper-extremity CRPS is not directly supported. Direct comparative trials between modalities are scarce, and no randomized trial has compared multimodal combination regimens against single-modality treatment.

Limitations of this review. This is a PRISMA-style pathway review, not a registered systematic review or de novo meta-analysis. The search was PubMed-centered by design; Embase and other subscription databases were not searched, and additional studies may exist. Screening was performed by a single reviewer without independent duplicate screening. Full-text extraction was limited to the 22 unique articles judged most useful after abstract and full-text review, which introduces potential selection bias. No new risk-of-bias scoring was performed beyond extracting the certainty judgments reported within the included reviews. No new pooled effect estimates were generated. The conclusions are deliberately conservative as a result.

A note on selection criteria. Throughout this review, we have offered candidate profiles for individual modalities. These are extrapolated from the entry criteria of the cited trials, not derived from validated predictive instruments. No trial has prospectively randomized patients by phenotype to individual modalities. The profiles should be applied as clinical heuristics rather than decision rules.

## Conclusions

CRPS remains one of the most challenging complications following upper-extremity trauma and surgery. Despite substantial advances in understanding its neurobiology, no single pharmacologic agent, interventional procedure, or rehabilitation technique has proven universally effective. Contemporary evidence supports an individualized, multidisciplinary approach centered on restoration of function. Surgeons should consider CRPS whenever postoperative recovery deviates from the expected course and apply the Budapest Criteria during serial examinations, establishing the diagnosis only after infection, tendon injury, hardware complications, persistent nerve compression, vascular disorders, and other surgically correctable conditions have been systematically excluded. Functional restoration should begin during the diagnostic process rather than after it and include occupational and physical therapy, edema management, desensitization, graded motor imagery, mirror therapy, patient education, and progressive return to limb use. Pharmacologic therapies, sympathetic blockade, ketamine infusion, and neuromodulation should be used as adjuncts that facilitate rehabilitation rather than replace it.

Unnecessarily prolonged immobilization, escalation of pharmacologic therapy in the absence of measurable functional improvement, attribution of persistent postoperative pain to CRPS before competing surgical complications have been excluded, and preoperative risk stratification based on demographic characteristics that have never been validated as predictive should be avoided. Success should not be defined solely by lower pain scores but by restoration of independence, return to work, and quality of life. Future research should prioritize high-quality multicenter randomized trials with standardized diagnostic criteria, validated functional outcome measures, and longer-term follow-up to clarify the optimal sequencing of rehabilitation, pharmacologic therapy, interventional procedures, and neuromodulation. Direct comparative trials between modalities, multimodal combination regimens versus single-modality treatment, and upper-extremity-specific neuromodulation studies remain needed. Continued investigation into biomarkers, advanced neuroimaging, and precision-medicine approaches may eventually refine patient selection beyond what is currently possible.

The greatest opportunity to improve outcomes lies not in increasingly complex interventions but in recognizing CRPS earlier, preventing it through careful surgical technique and structured rehabilitation, and coordinating multidisciplinary care before pain, immobility, and fear-avoidance become self-perpetuating. Even with this algorithm in mind and the best of intentions, I have found treating my CRPS patients an amazing challenge and a blessing. I cannot help everyone, but I can listen to every single patient. I can learn from every experience in the clinic and apply that to the next patient who comes to me with a hurt hand.

## Appendices

The PRISMA-style flow diagram (Figure 1) was generated using the companion Python script CRPS_PRISMA_flow_figure_code.py. The script stores the screening counts as a dictionary and draws the figure using matplotlib, so the figure can be regenerated and audited from code. No generative image tool was used.

Screening count dictionary used for the figure: records_identified_pubmed = 1471; duplicates_removed_database_stage = 0; title_citation_screened = 1471; first_pass_excluded = 283; potentially_relevant_after_first_pass = 1188; likely_relevant_after_staged_triage = 170; deferred_lower_priority_or_background = 1018; focused_triage_excluded = 75; core_records_to_abstract_review = 85; optional_secondary_background_records = 10; abstracts_reviewed = 85; core_included_after_abstract_review = 67; optional_background_after_abstract_review = 14; excluded_after_abstract_review = 4; full_text_priority = 56; top_full_text_set = 50; essential_first_download_subset = 30; pdfs_uploaded_for_full_text = 24; duplicate_full_text_pdfs = 2; unique_full_text_articles_reviewed = 22; unique_full_text_articles_excluded = 0; unique_full_text_articles_included = 22.

A master extraction file (CRPS_master_audit_final_22_articles.xlsx) records citation, study type, population, CRPS subtype, intervention, comparator, outcomes, key findings, limitations, and pathway relevance for each of the 22 included articles, and is available to editors and readers on request.
